# Hypertension secondary to renal hypoplasia presenting as acute heart failure in a newborn

**DOI:** 10.1186/s40885-019-0115-y

**Published:** 2019-05-01

**Authors:** Jena Deitrick, Kayle Stevenson, Daniel Nguyen, William Sessions, Vijay Linga, Tetyana Vasylyeva

**Affiliations:** Department of Pediatrics, Texas Tech University Health Sciences Center, Amarillo, TX USA

**Keywords:** Neonatal hypertension, Acute heart failure, Renal hypoplasia

## Abstract

**Introduction:**

Neonatal hypertension is defined as persistent systolic and/or diastolic blood pressures above the 95th percentile compared to other infants of similar gestational age and size. Neonatal hypertension is a rare condition, occurring in only 0.2–3.0% of neonates. The most common etiology of neonatal hypertension is renal vascular or parenchymal disease, and it is usually detected on routine examination in an asymptomatic child. However, it may present in a variety of manners, including acute heart failure, renal dysfunction, feeding difficulties, failure to thrive, tachypnea, apnea, lethargy, irritability, or seizures.

**Case presentation:**

A term female was born via repeat caesarean section with vacuum extraction. On day of life (DOL) 3, the baby presented to the emergency department with poor feeding and lethargy. Initial laboratory tests indicated severe metabolic acidosis and the patient was transferred to our neonatal intensive care unit (NICU). During the hospital stay, the patient had intermittently high blood pressures. An echocardiogram was ordered, which demonstrated a severely decreased ejection fraction of 33%, but no signs of coarctation of the aorta. The low ejection fraction and constellation of symptoms were consistent with the diagnosis of acute heart failure, so treatment with milrinone was initiated. Further labs demonstrated elevated renin and aldosterone, and a computed tomography scan showed right kidney hypoplasia with reduced perfusion. This suggested a renovascular etiology of hypertension causing the initial presentation of acute heart failure. The patient was started on enalapril and clonidine for blood pressure control and was discharged with a home blood pressure monitoring system. At 5 months of life, this patient was still on enalapril and amlodipine as well as home blood pressure monitoring.

**Conclusions:**

Acute heart failure is a rare presentation of neonatal hypertension, and prompt recognition and treatment for the underlying systemic hypertension is necessary to provide the best possible outcomes for patients. Due to the lack of sufficient evidence, treatment of hypertension in newborns is often anecdotal in nature. Further awareness of neonatal hypertension and research determining ideal methods of diagnosis and treatment would benefit physicians and their affected patients.

## Background

Neonatal hypertension is defined as persistent systolic and/or diastolic blood pressures above the 95th percentile compared to other infants of similar post-conceptual age, gestational age, and size [1]. This condition was first described over four decades ago as physicians recognized a correlation between umbilical artery catheters (UAC) and elevated blood pressures [2]. Usually, it is detected on routine examination in an asymptomatic child, but hypertension can also rarely present as acute heart failure [3, 4]. The most common etiology of neonatal hypertension is renal, but numerous other associations have been described in the literature [2]. While there is still great discourse over the diagnosis and treatment of neonatal hypertension, significant progress in defining, classifying, diagnosing, and treating this rare condition have been made. We present an unusual case of neonatal hypertension presenting as metabolic acidosis and acute heart failure as well as discuss current recommendations for diagnosis and treatment of neonatal hypertension.

**Fig. 1:**
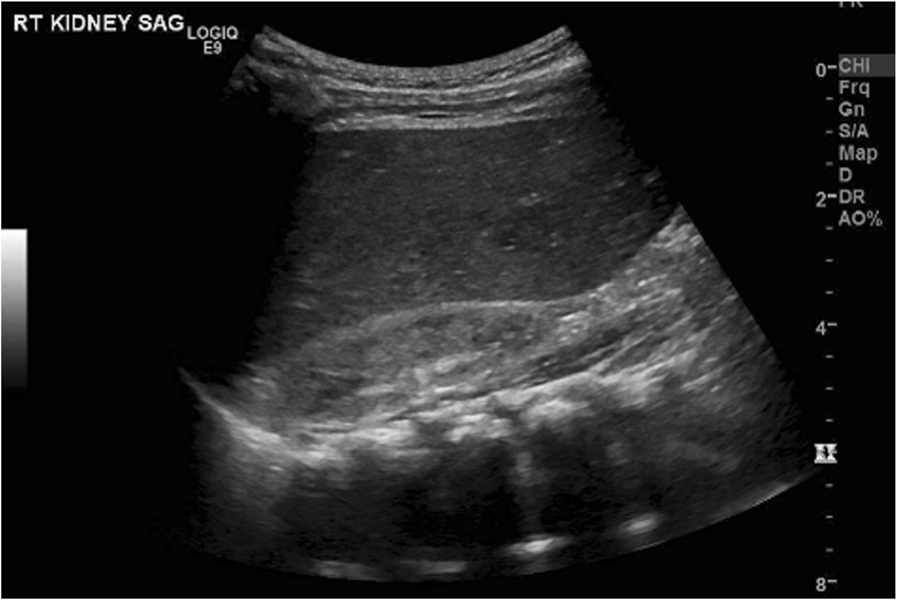
Renal Ultrasound of Left Kidney - 3.9 × 2.3 × 2.7 cm

**Fig. 2:**
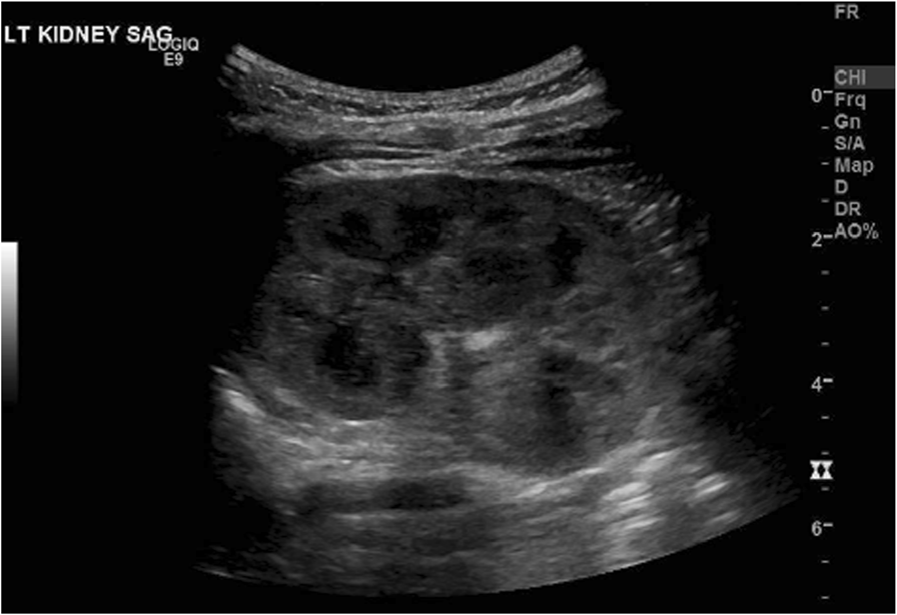
Renal Ultrasound of Right Kidney-5.1 × 2.5 × 2.9 cm

## Case report

A term female was born to a 25-year-old gravida-2 para-1 at 39 + 1 weeks gestation. The mother had a history of gestational hypertension in the previous pregnancy, but blood pressures were within normal limits during this gestation. After an uneventful pregnancy, the patient was born via repeat caesarean section with vacuum extraction. The birth weight was 2.66 kg and APGARs were 8 and 9 at 1 and 5 min, respectively. In the postnatal period, the baby had mild respiratory distress, resolved with blow-by oxygen. Physiologic jaundice was treated with phototherapy and the patient was discharged on DOL 2.

On DOL 3, the baby presented to the same hospital with poor feeding and lethargy. Initial laboratory tests indicated severe metabolic acidosis with an arterial blood gas (ABG) pH 6.9/ pCO_2_ 48/pO_2_ 50/HCO_3_^−^ 10.8/Base Deficit − 21. The patient was given sodium bicarbonate to resolve the acidosis and intubated prior to transfer. The patient was then transferred to a Level III NICU at our institution.

Upon admission, the differential diagnosis was inborn error of metabolism, shock due to sepsis and hypovolemia or cardiogenic etiology. Patient was kept nil my mouth (NPO), given intraveous fluids with dextrose10% water, and placed on mechanical ventilation. An high umbilical arterial catheter and umbilical venous catheter were placed in order to accurately monitor arterial and venous pressures, and obtain blood gases. Initial labs demonstrated white blood cells: 20.6 × 10^3^/mcL, hemoglobin: 14.8 g/dL, platelet 288 × 10^3^/mcL, neutrophils of 70% and lymphocytes of 18%, serum sodium:134 mmol/L, serum potassium: 6 mmol/L, serum bicarbonate: 15 mmol/L, serum blood urea nitrogen: 13 mg/dL, serum creatinine: 0.8 mg/dL, urine specific gravity: 1.004, serum lactic acid level: 7.4 mmol/L, which later worsened to 9.4 mmol/L and ammonia: 74mcmol/L. The patient’s blood pressure on admission was 119/70 mm of Hg in the right arm and 94/59 mm of Hg in the left arm. Empiric antibiotics (Acyclovir 20 mg/kg/dose q8H, Ampicillin 100 mg/kg/dose q8H, Meropenem 20 mg/kg/dose q8H) for suspected sepsis were initiated. However, cerebrospinal fluid studies, urinalysis, and blood cultures were later found to be negative, ruling out an infectious etiology.

The patient had intermittently high blood pressures during the hospital stay with systolic pressures in the 100’s mmHg, and diastolic pressures in the 70’s. An echocardiogram demonstrated mitral regurgitation, tricuspid regurgitation, and severely depressed left ventricular function with an ejection fraction of 33%. It also ruled out other structural cardiac anomalies, including coarctation of the aorta. The low ejection fraction and constellation of symptoms were consistent with the diagnosis of acute heart failure, so treatment with milrinone (1 mcg/kg/min) was initiated and the ejection fraction improved to 44%. The patient showed signs of improvement by DOL 5 with an ABG of 7.48/33.8/60/25/2 and was weaned off of mechanical ventilation. Trophic feeds were initiated; however this resulted in the patient becoming slightly acidotic with a pH of 7.32, so she was again made (NPO).

The patient was transferred to a Level IV NICU with a metabolic specialist, where complete work up demonstrated no inborn errors of metabolism. A CT scan showed right kidney hypoplasia with reduced perfusion, and further labs demonstrated an elevated renin of 194 ng/ml/hr. (Ref 2.0–35) and aldosterone of 1476 ng/dL (Ref 6.0–179.0). This suggested a renovascular etiology of hypertension causing the initial presentation of acute heart failure. After discharge, upon follow-up with the primary care physician (PCP), she was again found to have elevated blood pressures (data not available). The patient was admitted to another hospital and started on enalapril (1 mg/mL) and clonidine (0.1 mg/mL) for blood pressure control. The patient was discharged on this regimen with a home blood pressure monitoring system. She continued to have uncontrolled blood pressures (data not available) and was admitted again to our institution where amlodipine 0.1 mg/kg BID was added to her regimen, and adequate control was achieved. Repeat work up for pheochromocytoma with Urine 24 h vanillylmandelic acid was 0.5 mg/24 h, Urine 24 h normetanephrines 127 mcg/24 h, Urine 24 h metanephrine 12 mcg/24 h, were with in normal for age. Renal ultrasound: mild left renal pelviectasis. Otherwise, normal echogenicity of bilateral kidneys, Rt kidney was 3.9 × 2.3 × 2.7 cm and Left Kidney 5.1 × 2.5 × 2.9 cm (Fig. 1). Renal artery duplex ultrasound: No evidence of hemodynamically significant disease bilaterally. At subsequent follow-up appointments, the clonidine was discontinued, and adequate blood pressures were maintained. At 5 months of life, patient was still on enalapril and amlodipine as well as home blood pressure monitoring. Follow-up renal ultrasound: The right kidney is small 4.47 × 1.81 × 1.27 cm and left was 6.25 × 3.04 × 3.49 cm (Fig. 2). There is no renal collecting system obstruction identified. Her blood pressures are moderately controlled, and her growth and development were appropriate for post-conceptual age.

## Discussion

Neonatal hypertension is a rare condition, occurring in 0.2–3.0% of neonates [1, 3, 5, 6]. It is associated with prematurity, very low birth weight, UAC, antenatal and maternal factors, renal artery stenosis, renal parenchymal disease, bronchopulmonary disease, patent ductus arteriosus, coarctation of the aorta, genetic disorders/syndromes, endocrine disorders, tumors, and medications [1–7].

The most common risk factor for neonatal hypertension is UAC and 6–25% of infants with a UAC will have an associated renal venous thrombosis [1, 3, 7]. The resulting renovascular compromise causes activation of the renin-angiotensin-aldosterone system, which results in increased blood pressure. The placement of UACs has been divided into “high” and “low” based on the location of the end of the catheter. “High” placement refers to catheters ending at the T6-T9 level in the abdominal aorta, above the celiac trunk. “Low” placement refers to catheters ending at the L3-L4 level of the aorta, below the renal arteries. While “high” placement of UACs results in lower rates of thrombus formation, it does not result in lower rates of hypertension [3]. Our patient had a UAC placed in order to monitor arterial pH and blood pressure, but there was no evidence of thrombus formation secondary to UAC placement.

Neonatal hypertension is associated with maternal hypertension, maternal abuse of heroin or cocaine, and antenatal steroid administration [3, 7]. Maternal hypertension leads to increased sensitivity to endothelin and cytokine-induced endothelial cell damage [1]. Antenatal steroids may cause irreversible reduction in nephron number and increase the receptor density of the renin-angiotensin-aldosterone system [1]. Postnatal medication-use in the infant, including dexamethasone, aminophylline, phenylephrine ophthalmic drops, and long-term total parenteral nutrition, are also associated with neonatal hypertension [5]. Premature infants have decreased nephrogenesis in utero, making them vulnerable to hypertension, and their blood pressure rises at a greater rate compared to that of term infants [3]. Term infants are often diagnosed earlier and are more likely to present with hypertension resistant to medical interventions [8].

Aside from the aforementioned associations, the most common cause of neonatal hypertension is renal disease. This includes poor renal flow from renal artery stenosis, congenital renal anomalies, and acquired parenchymal disease. Renal artery stenosis is caused by idiopathic arterial calcification, congenital rubella infection, fibromuscular dysplasia, and mechanical compression [3]. Congenital renal anomalies, including polycystic kidney disease, pelvic kidney, hypoplastic or absent kidney, severe vesicoureteral reflux, and posterior urethral valves, typically present within the first year of life [1, 3, 5, 7]. Renal disease may be acquired, such as in the setting of acute renal failure, acute tubular necrosis, and interstitial nephritis. In all cases, signs of renal dysfunction can be seen in the blood pressure as well as electrolyte and creatinine levels.

Neonatal hypertension is commonly found in asymptomatic neonates, but it can also present as feeding difficulties, failure to thrive, tachypnea, apnea, lethargy, irritability, and, in very rare cases, acute heart failure [9–13]. In addition to monitoring blood pressure, an echocardiogram should be done to rule out structural heart diseases, including obstructive cardiac defects [12]. In patients whose hypertension has led to acute heart failure, they may demonstrate mitral regurgitation as well as systolic dysfunction, demonstrated by a decreased ejection fraction [12]. Our patient demonstrated both mitral regurgitation as well as a severely decreased ejection fraction. In addition to treating the systolic dysfunction with ionotropes, these patients must be considered candidates for anti-hypertensives [12].

Diagnosis of neonatal hypertension is challenging due to the inaccuracy of methods of obtaining blood pressure in neonates as well as the constantly changing blood pressure of newborns. The accuracy of oscillometric devices depends on the size of the infant [3]. Furthermore, the activity state (eg. crying, sleeping, moving) and position of the infant can greatly impact the measurements. Supine blood pressure readings are higher than prone readings, and blood pressure can increase up to 20 mmHg while feeding and increase 10 mmHg while sucking on a pacifier [3]. Intra-arterial blood monitoring (IBP), utilizing the umbilical, radial, or posterior tibial artery, is currently the gold standard for neonatal blood pressure monitoring, but they can lead to thrombosis or infection [2].

Diagnosis is further obscured by the fact that neonatal hypertension typically has a delayed presentation. The average age of diagnosis for neonatal hypertension is DOL 5 to corrected age 2 months [1, 3, 4]. Therefore, many hypertensive infants may be missed if they are discharged prior to presentation. Finally, while diagnosis cut-offs depend on population data of “normal” infantile blood pressures, these published values were created using a small number of observations, and may not be an accurate depiction of normal blood pressure measurements in neonates [3]. Overall, there is great room for improvement in standardizing the diagnosis of neonatal hypertension.

Workup of neonatal hypertension depends on the most likely cause of elevated blood pressure. Prenatal and birth history can reveal significant associated factors, but further workup is typically required. Chest X-ray can be used in the setting of lung disease, such as bronchopulmonary dysplasia, and echocardiography is used to diagnose suspected cardiovascular anomalies. Workup of suspected renal disease commonly includes urinalysis, complete blood count, comprehensive metabolic panel, renal ultrasound with Doppler, and renin/aldosterone levels with ratios. One study found that aldosterone levels were elevated in 60% of patients with neonatal hypertension while renin was elevated in 33% of patients [1]. While this is indicative of renal disease, renin is typically high in infancy, especially in premature infants [7]. If renal ultrasound with Doppler is suggestive of renal artery stenosis, it must be followed by an arteriogram, which is the gold standard for diagnosis [7]. Other common tests for the workup of neonatal hypertension include thyroid studies, cortisol levels, calcium levels, abdominal/pelvic ultrasound, and urine homovanillic acid and vanillylmandelic acid [3]. After a thorough workup has identified a cause of the neonate’s hypertension, treatment can be tailored for the specific cause.

While treatment of neonatal hypertension remains widely unstudied, it relies heavily on expert advice and research pertaining to the treatment of childhood, adolescent, and adult hypertension. Common medical interventions include diuretics, angiotensin converting enzyme (ACE) inhibitors, calcium channel blockers, beta blockers, and alpha antagonists [9]. ACE inhibitors and beta-blockers have the most evidence of effectiveness in lowering blood pressure in neonates [10]. Calcium channel blockers currently have the least amount of evidence of their efficacy in neonatal hypertension [10]. The drug of choice typically depends on the severity of hypertension. Malignant hypertension, which is above the 99th percentile, is treated with nicardipine, labetalol, esmolol, nitroprusside, and/or fenoldopam [2]. Moderate hypertension is treated with diuretics, propranolol, or hydralazine [2]. Thiazides are the drug of choice for mild hypertension in infants [11]. Commonly used oral medications for chronic management include isradipine, amlodipine, nifedipine, minoxidil, and captopril [3]. Due to the lack of evidence, there is not yet a consensus on the most effective and safe treatment for hypertension in neonates and infants.

The prognosis for infants with hypertension depends on the underlying cause. The majority of cases resolve, and only 15% of patients continue to require medications by 6 months of age [1, 5]. Persistent hypertension can result in various presentations and complications, including congestive heart failure, cardiogenic shock, renal dysfunction, hypertensive retinopathy, seizures, and increased risk of developing hypertension later in life [3]. Improving accurate diagnosis and treatment of neonatal hypertension can prevent the consequences of persistent hypertension and lead to better outcomes for these patients.

## Conclusion

This is an unusual case of hypertension in a newborn presenting as acute heart failure. Prompt recognition of systemic hypertension as the cause of systolic dysfunction is necessary to facilitate early treatment and prevention of further complications of severely elevated blood pressures. In addition to furthering awareness of this uncommon neonatal condition and various presentations, we assert that there is great need for further research to support efficacy, safety, and standardization of therapeutic interventions for neonatal hypertension.
